# Uncertainty, Cognitive Control and Theta‐Band Activity: A Relationship That Depends on Metacontrol Requirements

**DOI:** 10.1002/hbm.70333

**Published:** 2025-09-23

**Authors:** Seema Prasad, Nasibeh Talebi, Paul Wendiggensen, Moritz Mückschel, Bernhard Hommel, Christian Beste

**Affiliations:** ^1^ Cognitive Neurophysiology, Department of Child and Adolescent Psychiatry Faculty of Medicine, TU Dresden Dresden Germany; ^2^ School of Psychology Shandong Normal University Jinan China; ^3^ German Center for Child and Adolescent Health (DZKJ), Partner Site Leipzig/Dresden Dresden Germany

**Keywords:** cognitive control, curiosity, effective connectivity, posterior cingulate cortex, theta band activity, uncertainty

## Abstract

Cognitive control is assumed to be intricately linked to theta band activity. Situations that involve high uncertainty are said to trigger a need for cognitive control, which is reflected in greater theta activity. We examined whether theta band activity is similarly implicated in cognitive control processes when uncertainty is likely to trigger curiosity—a motivational state that makes people explore their environment. We investigated this in a sample of *N* = 41 healthy human adults by manipulating target‐related uncertainty in a Posner cueing task. Time–frequency and beamforming approaches were applied to analyse the oscillatory dynamics and their sources. Effective connectivity analysis was done to examine how information transfer is modulated by uncertainty. Behavioural results showed greater sensitivity to task‐irrelevant cues under high uncertainty. Importantly, there was no theta band activity in the posterior cingulate cortex under high compared to low uncertainty. Effective connectivity analyses also showed weaker connections between inferior parietal lobule and posterior parietal cortex under high uncertainty. Alpha band activity in the temporo‐parietal junction under high uncertainty indicated an effect of uncertainty on early attentional filtering. These results indicate that high uncertainty is not always associated with increased theta band activity. We discuss possible explanations of this finding including that uncertainty may trigger different (meta) control policies which could be associated with distinct oscillatory dynamics. These findings have implications towards our understanding of ‘need for control’ and the situations that trigger it.

## Graphical abstract

Uncertainty is typically linked to the need for cognitive control reflected in greater theta band activity. We manipulate uncertainty as a trigger for curiosity in a spatial cueing paradigm. Time–frequency and DICS beamforming results show that uncertainty is not always linked to greater theta band activity.

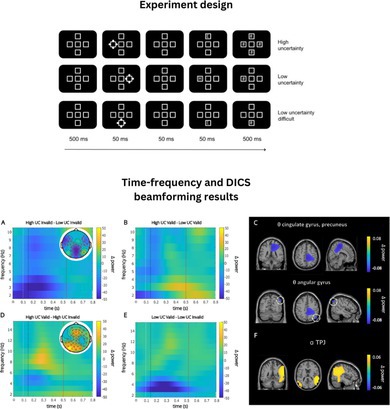

## 1 Introduction

The neurophysiological processes supporting goal‐directed actions have been intensely studied. A canonical finding is that especially (medial frontal) cortex theta band activity (TBA) supports processes relevant for action control (Beste et al. 2023; Cavanagh and Frank 2014). The medial frontal TBA is often proposed to reflect a ‘surprise signal’, which indicates that something needs to be done but does not code what this should be (Cavanagh and Frank 2014). TBA usually increases in situations occurring rarely and (therefore) imposes high demands on cognitive processes (Cohen 2014a; Ullsperger et al. 2014). Importantly, TBA is modulated by the ‘uncertainty’ that specific response options occur or that specific information will be obtained in the future (Dippel et al. 2016, 2017; Monsalve et al. 2018; van Wingerden et al. 2010) and some evidence suggests that TBA codes predictions of upcoming events (Arnal and Giraud 2012; Buzsáki and Draguhn 2004). In conceptions of TBA as a ‘surprise signal’ for control, uncertainty is seen as being undesired as it triggers the need to increase effortful cognitive control. For instance, Dippel et al. (2017) observed greater TBA as the frequency of no‐go trials decreased, suggesting that exerting cognitive control becomes more demanding as events that require inhibition become rare or uncertain.

However, uncertainty is not a unitary concept. While some approaches outlined above view uncertainty as a trigger to monitor and adjust behaviour in line with top‐down goals, others view uncertainty as a trigger for curiosity (Gottlieb and Oudeyer 2018; Kidd and Hayden 2015; Van Lieshout et al. 2021). Curiosity is the intrinsic drive to seek more information ‘for its own sake’ (Berlyne 1960). It is not driven by external rewards or benefits, but the reward consists of the information gained out of exploration (see FitzGibbon et al. 2020 for a review; Kang et al. 2009; Kobayashi and Hsu 2019). Self‐reported curiosity activates the same reward network in the brain that is commonly associated with extrinsic reward cues (Kang et al. 2009; Kobayashi and Hsu 2019). Curiosity is typically marked by a reduced impact of top‐down goals and being more open to a wide range of information. In line with this, it has been seen that irrelevant faces presented during a curiosity‐inducing trivia task are recalled better on high curiosity trials compared to low curiosity trials (Gruber et al. 2014; Gruber and Ranganath 2019). It is not clear whether and to what degree these different conceptualizations of uncertainty (as a trigger for goal‐directed control vs. a trigger for curiosity) rely on the same control processes. It is possible that the type of cognitive control needed depends on the type of uncertainty that is encountered (Hommel and Colzato 2017). Given this and that TBA has been shown to differentiate between distinct cognitive control strategies (Cavanagh et al. 2012; Eisma et al. 2021), it is reasonable to ask whether TBA is similarly involved when uncertainty is linked to curiosity.

In the current study, we used an EEG experiment to investigate how uncertainty affects behaviour in a spatial cueing task (Shiu and Pashler 1994). In this paradigm, the location of task‐irrelevant peripheral cues is known to influence responses to a target (Chica et al. 2014; Posner 1980). To manipulate uncertainty, we varied the number of masks presented after the target: either a single mask or four masks. This approach, adapted from previous work (Prasad and Hommel 2024; Shiu and Pashler 1994), modulates how precisely the target location can be identified. In the low‐uncertainty condition, a single mask always appeared at the target location, making the target's position fully predictable. In contrast, the high‐uncertainty condition included four masks, one at each possible location, obscuring the target's position. To control for potential differences in task difficulty, we included an additional block with the single‐mask condition but increased overall task difficulty. We tested whether the influence of peripheral cues on behaviour was affected by this uncertainty manipulation. We hypothesised that repeated exposure to a given mask condition would induce a sustained state of low or high uncertainty (high UC) across a block of trials, influencing cognitive control processes engaged during the task.

While we focused on TBA, we also examined the modulation of alpha band activity (ABA). The reasons are that TBA is modulated by ABA during response selection (Beste et al. 2023; Cavanagh and Frank 2014; Rawish et al. 2024; Wendiggensen et al. 2023) and that ABA is central to the filtering of incoming information and attentional processes (Klimesch 2012; Herrmann and Knight 2001) that are captured by the experimental approach used in this study. We used EEG‐beamforming analyses to examine which functional neuroanatomical structures are associated with modulations in TBA and ABA. Based on previous findings, it is most likely that besides medial frontal cortices, superior and inferior parietal regions are involved, since these regions are related to perception and action (Gottlieb 2007) and, particularly, so when information to update internal representations of the environmental context in order to initiate appropriate actions is unexpected (Geng and Vossel 2013). In further exploratory analyses, we used non‐linear causal relationship estimation by artificial neural networks (nCREANN) (Elmers et al. 2024; Talebi et al. 2019) to examine whether and how information transfer between the involved functional neuroanatomical structures is modulated. We did not look into other frequency bands (e.g., beta/gamma) as there was no clear hypothesis linking uncertainty and cognitive control to these frequency bands. Thus, we only focused on theta and alpha bands as we had theoretical reasons based on existing literature.

## 2 Materials and Methods

### 2.1 Sample

*N* = 41 healthy adults (19 male, 22 female, mean age = 27 years, SD = 4) participated in this study. The sample size was determined based on an earlier study (Shiu and Pashler 1994) from which the design was adapted. There were *N* = 12 participants in Shiu and Pashler (1994, Experiment 1). Cohen's standardised difference scores (*d*_z,_ Cohen 1988) estimated using the reported paired‐sample *F* test values and sample sizes were 1.01. The calculations were based on results reflecting differences between cue valid and invalid trials. The power analysis (using ‘pwr’ package in R) yielded a sample size of 10 for a desired power of 0.8 with the confidence level set to 0.05. A larger sample was selected because we had an additional experimental condition. This sample size is similar to the *N* in several other EEG studies with similar methodology published recently (Brilliant et al. 2024; Magosso and Borra 2024; Pscherer et al. 2023; Rawish et al. 2024). Further, we had a large number of trials (768) for each participant, contributing to the study's power. All participants were in the age range of 18–35 years and reported no neurological or psychiatric disorders. Written informed consent was taken from all participants. One participant's data was excluded from all analyses because the stimulus and response markers for the EEG recording were missing due to technical errors. Behavioural and EEG data from the remaining *N* = 40 participants were included in the data analyses. The institutional ethics committee of TU Dresden approved the study.

### 2.2 Task

**FIGURE 1:**
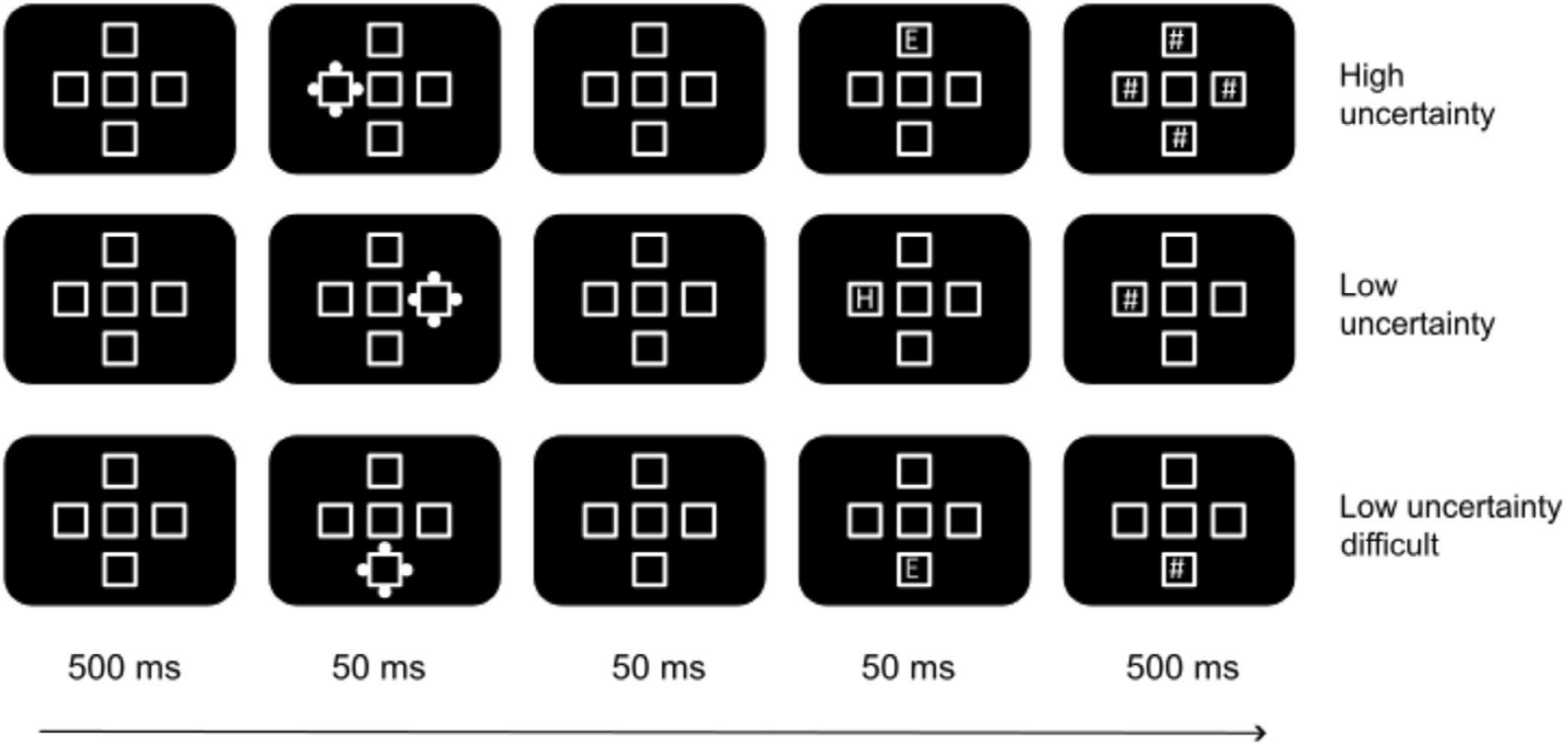
Sequence of events on a sample trial in all three conditions. The cue and the target appeared in any one of the four placeholders around the central placeholder. The figure shows an example invalid trial (cue and target appear at different locations) in the high and low UC conditions. An example valid trial (cue and target appear at the same location) is shown for the low UC difficult condition.

The task is shown in Figure 1.

Each trial started with the presentation of a central square (2.5°) surrounded by four squares arranged in the form of a plus sign (top/bottom/left/right) on a screen with a refresh rate of 60 Hz. We do not report other physical properties of the screen, such as brightness, because we consider them relevant only in paradigms involving tightly controlled psychophysics methods. Each of the four squares was at a distance of 1.25° from the central square. The task design was borrowed from Prasad and Hommel (2024) who reported two experiments with the same task administered online. The spatial arrangement of the visual objects in these studies was adopted from previous studies (Prasad et al. 2021, 2022; Ruthruff and Gaspelin 2018). The squares served as placeholders and were in white on a black background. After 500 ms, the ‘cue’ was presented for 50 ms in the form of four filled white dots around one of the four squares. After 50 ms, the target letter (‘E’ or ‘H’ white colour, 2° height) was presented inside one of the four squares for 50 ms. Thus, the targets were presented 100 ms after cue onset. Following this, the symbol ‘#’ was shown for 500 ms to mask the visibility of the target. On half of the trials, the ‘#’ symbol was shown inside all the four squares (four‐mask trials with high UC). On the other half, the symbol was shown only inside the target square (single‐mask trials with low uncertainty [low UC]). Following the presentation of the masks, the participants were asked to identify the target letter (‘E’ or ‘H’) and press the corresponding key on the keyboard. The trial ended only after a response was made. The target could appear either at the cued location (‘valid’ trials) or at a different location (‘invalid’ trial). Since our objective was to make the cue nonpredictive of the target location, there were 25% valid trials and 75% invalid trials in the experiment. There was a total of 768 trials divided into three blocks. There was one block of 256 trials with low UCand another block of 256 trials with high UC. A third block of trials was identical to the low UC condition except that the target letter was presented in grey (#737380) to increase the difficulty of the task by reducing the contrast between the target letter and the background (‘low UC difficult’ condition). Our main comparison is between high and low UC conditions. However, it is possible to attribute any differences found between high and low UC to a possible increase in difficulty in the high UC condition rather than the increase in uncertainty. To address this, we kept the uncertainty low, but only increased the task difficulty in one block to rule out confounding explanations. The trials were blocked in each mask condition to induce a sustained state of curiosity in a block of trials. The block order was randomised.

There were 24 practice trials at the beginning of the experiment where feedback was given on every trial with an incorrect response. In the main experimental blocks, participants were given self‐paced breaks after every 64 trials. During this break, they were also given feedback on their mean response time (RT) and accuracy in the preceding block.

### 2.3 Behavioural Data Analyses

We first checked if all participants had accuracy greater than 55% (cut‐off criterion borrowed from Prasad and Hommel (2024)). All participants cleared this criterion. The signal detection measure *d*′ was calculated for each condition and participant. The target letter ‘E’ was considered as signal and ‘H’ as noise (this assignment is arbitrary and can be reversed). Correct responses to ‘E’ counted as Hits and incorrect responses to ‘H’ counted as false alarms. The *d*′ score was calculated as the difference in *z*‐transform values of hit rates and false alarm rates. RTs were analysed after discarding trials with incorrect responses (21%) and RTs faster than 100 ms and slower than 2000 ms (2.1%). Repeated measures ANOVA was conducted on *d*′ and mean RTs with UC Condition (high UC, low UC and low UC difficult) and Validity (valid, invalid) as factors. Results from the repeated measures ANOVA on accuracy are reported in the Supporting Information (Analysis in Data S1).

### 2.4 EEG Pre‐Processing

During the experiment, EEG data was recorded using QuickAmp and BrainAmp amplifiers (Brain Products GmbH, Gilching, Germany) and a 60 channel Ag‐AgCl equidistant electrode setup. The reference electrode was set to Fpz. Recordings were conducted at a sampling rate of 500 Hz, which were downsampled to 256 Hz during later pre‐processing. Electrode impedances were kept below 5 kΩ. After recording, offline pre‐processing was done using automagic (Pedroni et al. 2019) within EEGLAB (Delorme and Makeig 2004) on Matlab 2022a (The MathWorks Corp.) using the concatenation of steps also used in previous studies by our group (e.g., Koyun et al. 2023; Pscherer et al. 2023; Yu et al. 2023). Automagic has been extensively used both by our group and other researchers (the paper introducing Automagic published in *NeuroImage* has been cited 240 times). Most EEG researchers would agree that there are no golden rules when it comes to EEG pre‐processing. Most often, individual PIs or groups decide on a pipeline based on their experience and subjective criteria. The biggest advantage of Automagic is that it offers a transparent, standardised procedure for pre‐processing that also helps with replicability. The first step in Automagic consisted of the removal of flat channels. The data were then re‐referenced to an average reference primarily because we aimed to perform source reconstruction following the time–frequency analyses. Average referencing is typically recommended for source reconstruction techniques as it reduces the forward model error introduced by localization inaccuracies of the electrodes' positions (Westner et al. 2022). After this step, the PREP pre‐processing pipeline (Bigdely‐Shamlo et al. 2015) was applied to remove the line noise at 50 Hz using a multitaper algorithm. Next, clean_rawdata() was applied, which first detrends the EEG data using an FIR high‐pass filter of 0.5 Hz. Flat‐line, noisy and outlier channels were detected and removed. Epochs showing abnormally strong power (> 15 standard deviations relative to calibration data) were reconstructed using artifact subspace reconstruction (ASR, Mullen et al. 2013). Calibration was done based on the default method in the clean_asr function, where a subset of clean data was identified from the given recording using the Statistics toolbox. Time windows that could not be reconstructed were removed using the WindowCriterion parameter in the clean_rawdata pipeline with the default setting (0.25). This means that time windows in which more than 25% of the channels had contaminated data (within that time window) that could not be reconstructed were then removed. A low‐pass filter of 40 Hz was applied. EOG artifacts were removed using a subtraction method (Parra et al. 2005). Muscle, loose electrodes and remaining eye artifacts such as blinks and saccades were automatically classified and removed by using an independent component analysis (ICA) based multiple artifact rejection algorithm (MARA, Winkler et al. 2011). Components containing cardiac artifacts were identified using ICLabel (Pion‐Tonachini et al. 2019) and removed consecutively. Finally, all channels that were removed by Automagic were interpolated using a spherical method. After the pre‐processing, segments were created for time‐locked data with the onset of the cue for each valid and invalid trial separately in each of the three UC conditions. The segments were 4000 ms long and began 2000 ms before cue onset. Long segments were chosen to allow for reliable quantification of low oscillatory activity (Beste et al. 2010; Cohen 2014b). Longer segments also ensure that edge effects do not contaminate the time window of interest. Since we were interested in the 0–1000 ms time window after cue onset, we chose a sufficiently long buffer zone before and after the window of interest to avoid edge artifacts. Next, a baseline correction was applied using the time window starting 200 ms before cue onset. This duration was chosen as it is generally recommended to have a short baseline (e.g., 200–400 ms) since having a longer baseline could result in contamination by other cognitive processes (Handy 2009; Luck and Kappenman 2013).

### 2.5 EEG Time–Frequency Decomposition and Beamforming

Activity in the theta (4–7 Hz) and alpha (8–12 Hz) bands was analysed using time–frequency decomposition methods with a Morlet parameter of 5 cycles, with a spectral bandwidth of 3 Hz and a time resolution of 53 ms using FieldTrip (Oostenveld et al. 2011). Average alpha and theta power were calculated for each time point at each electrode in the time window from 0 to 1000 ms starting from cue onset. The power values were compared between valid and invalid trials for each of the three UC conditions (high UC, low UC and low UC difficult) using cluster‐based permutation tests (CBPTs). The objective was to identify the set of electrodes showing significant differences in power between valid and invalid trials and to further examine if the differences between valid and invalid trials were modulated by the UC conditions. In the first step of a CBPT, paired *t* tests are conducted at each electrode. If at least two pairs of neighbouring electrodes show a significant difference (*p* < 0.05), they are considered part of a sample cluster. The sum of the *t* values in each cluster is taken to form the cluster‐level statistics. In the next step, the significance probability is calculated using the Monte Carlo method in which 1000 random draws of trials are tested for significant differences to approximate the reference distribution (Mückschel et al. 2016). The proportion of randomly drawn trials that show a larger test statistic than the observed results gives the *p*‐value. If a cluster reaches a *p*‐value below 0.05, it is considered to indicate significant differences in activity (at the given cluster of electrodes) between the conditions being compared. This procedure of CBPTs was repeated for each of the two frequency bands (theta and alpha). We followed the recommendation of the Fieldtrip toolbox tutorial to compute statistics as *t* tests between differences rather than compute *F* statistics with an ANOVA because the *t* and *F* distributions are expected to lead to different *p* values. The *F* statistics are also usually more conservative, meaning that differences between conditions are less likely to be found. Finally, given that most previous studies have used t values, it is recommended to use them to enable comparability with previous studies. Thus, separate CBPTs were performed for each condition. A Bonferroni correction method was applied to correct for multiple comparisons. A grand average was computed by averaging across all participants' data, which was then used to plot the time–frequency decomposition of spectral power at the significant cluster of electrodes showing differences in activity.

Next, we sought to identify the clusters of voxels associated with theta and alpha activity using the dynamic imaging of coherent sources (DICS method, Gross et al. 2001). This method has been previously applied by our group in several studies (Adelhöfer and Beste 2020; Dippel et al. 2017; Ghorbani et al. 2024; Pscherer et al. 2023; Rawish et al. 2024). It is worth noting that all source‐localization methods based on sensor data come with shortcomings. As is well known, EEG recordings have high temporal precision and lower spatial resolution. However, given the benefits of conducting both time–frequency analyses and source reconstruction together, some tradeoffs are unavoidable. DICS beamforming is an advanced analysis method that is a robust method to reconstruct sources compared to other traditional source localization methods (Westner et al. 2022). Therefore, we consider our spatial estimates to be as reliable as possible with the EEG methodology. Further, our findings are not interpreted at mm resolution but only in terms of general region. It is possible to question whether the DICS fully captures the complexity of brain activity. No model can fully capture the complexity of brain dynamics, as models are only a reasonable approximation of what is measured. We were specifically interested in the oscillatory dynamics and their neural basis, to which purpose DICS is commonly used by many researchers. It is possible to use alternate methods that could have other benefits (and costs). Since the purpose of this study was not to compare the suitability of source localization methods, we made an informed choice based on existing research and our past experience.

DICS beamforming was conducted in the time‐window between 0 and 600 ms after cue onset for alpha and theta band separately using common spatial filters calculated from the cross‐frequency spectra of a Fast Fourier Transformation (FFT) on the averaged power. The localization of activity was projected into a source space onto an equally spaced grid created from the forward model template of the FieldTrip toolbox, which is based on the standard Montreal Neurological Institute (MNI). For each UC condition, the power values of the valid and invalid trials were extracted separately and compared. Clusters of voxels showing significant differences between valid and invalid trials in each UC condition were identified using CBPTs as described earlier. An additional thresholding method was applied where voxels with the Top 5% activity were also selected. These clusters of voxels were considered the regions of interest (ROI) for further connectivity analysis.

### 2.6 Effective Connectivity Analysis

We examined the effective connectivity between the anatomical regions responsible for the effect of uncertainty in the theta frequency band. Two distinct ROI were created for the connectivity analysis: ‘CG’ which predominantly included the cingulate cortex, precuneus and paracentral regions and ‘AG’ which included the angular gyrus and the inferior parietal lobule (IPL). This analysis was not performed for the ABA as DICS beamforming revealed only a single large cluster of activity. To prepare the data for the connectivity analysis, a Hamming Windowed Sinc FIR filter was applied to pre‐processed EEG data to filter signals for the theta frequency band. This returned a time series with the same structure as the pre‐processed EEG data for the theta frequency band. The resulting time series were then segmented for valid and invalid trials of all three UC conditions and the time courses of their underlying sources were extracted in the next step. For each ROI determined through DICS beamforming and CBPT, we applied the linearly constrained minimum variance (LCMV) beamformer (Van Veen et al. 1997) for the theta frequency band to construct the source activity from sensor‐level data. This was done for the invalid trials of high and low UC conditions as we expected the two UC conditions to differ primarily on invalid trials and aimed to focus the connectivity analyses on these two conditions. A leadfield matrix was calculated using a Fieldtrip template ‘standard_bem’ as head model. Next, a common spatial filter was computed by concatenating the averaged data of both conditions. This common spatial filter was used to reconstruct the time course of the source activity (“source signals’) in each condition.

To analyse the effective connectivity in the present study, we utilised the machine learning‐based approach called nCREANN (Elmers et al. 2024; Talebi et al. 2019). The nCREANN employs an artificial neural network (ANN) to implement a non‐linear multivariate autoregressive (nMVAR) model of the source signals and assess the interactions between them. The nMVAR modelling captures both linear and non‐linear dynamics of the brain system, which has been shown to be crucial for the organisation of information flow across cortical regions (Kodama and Galán 2019; Yang et al. 2018). Within this model, the current sample of each signal is expressed as a (non)linear function of its past values and past values of other signals, enabling an inference of temporal causality (an effect causes the future).

For a given multivariate time series $x\left(n\right)\in {\mathbb{R}}^{M}$ of length *L*, a non‐linear MVAR model of order p is defined as

(1) $$x\left(n\right)=f\left(x_{p}\right)+\sigma \left(n\right)$$

where $x_{p}={\left[x_{1}\left(n-1\right),x_{2}\left(n-1\right),\cdots ,x_{M}\left(n-p\right)\;\right]}^{T}$ is the vector of p past samples of (*M*) time series. The noise vector, $\sigma \left(n\right)={\left[\sigma_{1},\sigma_{2},\ldots ,\sigma_{M}\;\right]}^{T},$ is the model residual and the non‐linear function $f\left(.\right)$ quantitatively describes how the p previous samples cause the future values. In the nCREANN method, the function f is divided into linear and non‐linear part

(2) $$f=f^{\mathrm{Lin}}+f^{\text{NonLin}}$$

and based on the $f^{\mathrm{Lin}}$, the linear connectivity $\left({\mathrm{lC}}_{i\to j}\right)$ is computed as the linear impact of *i*th region on the *j*th region and based on the information embedded within $f^{\text{NonLin}}$, the non‐linear connectivity, $\left({\mathrm{NC}}_{i\to j}\right)$, is inferenced to establish the extent of the non‐linear causal effect of $x_{i}$ on $x_{j}$.

In the present study, the nCREANN was applied to the time courses of the LCMV‐derived sources in the invalid trials of the high and low UC conditions. The data points of the trials in the time interval [0–1000] ms of the stimulus onset were considered for the connectivity analysis. For training the network, all of the single‐trial source signals were concatenated in order to have a sufficient data length. The optimum model order (*p* = 10) was estimated using Akaike and Schwartz criteria (Neumaier and Schneider 2001) and was considered the same for all subjects in both conditions.

A Multilayer Perceptron neural network with one hidden layer and 10 hidden neurons was trained. The network's input was the $x_{p}$ and it tries to predict $x\left(n\right)$ as its output. The training algorithm was gradient descent error back‐propagation (EBP) with momentum (*α*) and adaptive learning rate (*η*). The early stopping technique was applied for the sake of generalisation. The 10‐fold permuted cross validation technique was conducted and in each fold the data was divided into 80% training, 10% validation and 10% testing sets. The network parameters were updated in the ‘incremental’ mode (each time an input is presented to the network), with random initial parameters in the range of [−0.5, 0.5].

The network performance and the goodness of fit of the nMVAR model were assessed using mean square error (MSE) and the coefficient of determination criteria for the training and test data. Coefficient of determination (or *R* ^2^) is a statistical metric used in regression models that determines their validity. If a model fits the data well, its corresponding *R* ^2^ value will be close to 1. MSE is the most widely used measure to assess a network's performance. A properly trained network exhibits not only small‐training error, but also its test error falls within the range of training error. Furthermore, the similar *R* ^2^ for the training and test sets emphasises the network's appropriate generalisation.

The significance of the resulting connectivity values was evaluated assuming a randomisation test with generation of 100 data points based on time‐shifted surrogate technique (Papana et al. 2013). This method destroys any causal effect between the signals without changing the dynamics of each time series. For applying nCREANN to the surrogate data, the network parameters were set exactly the same as those used for original data. A 90% confidence interval was considered as a threshold to determine the significance of a connection. Connections that were below this threshold were not considered for further statistical analyses. Paired *t* tests were conducted comparing the linear connectivity values between high UC invalid and low UC invalid trials for each connectivity direction. Similar comparisons were made for the non‐linear connectivity values using the Wilcoxon signed rank test. A non‐parametric test was used for the non‐linear connectivity values as the data violated the normality assumption.

## 3 Results

### 3.1 Behavioural Data

**FIGURE 2:**
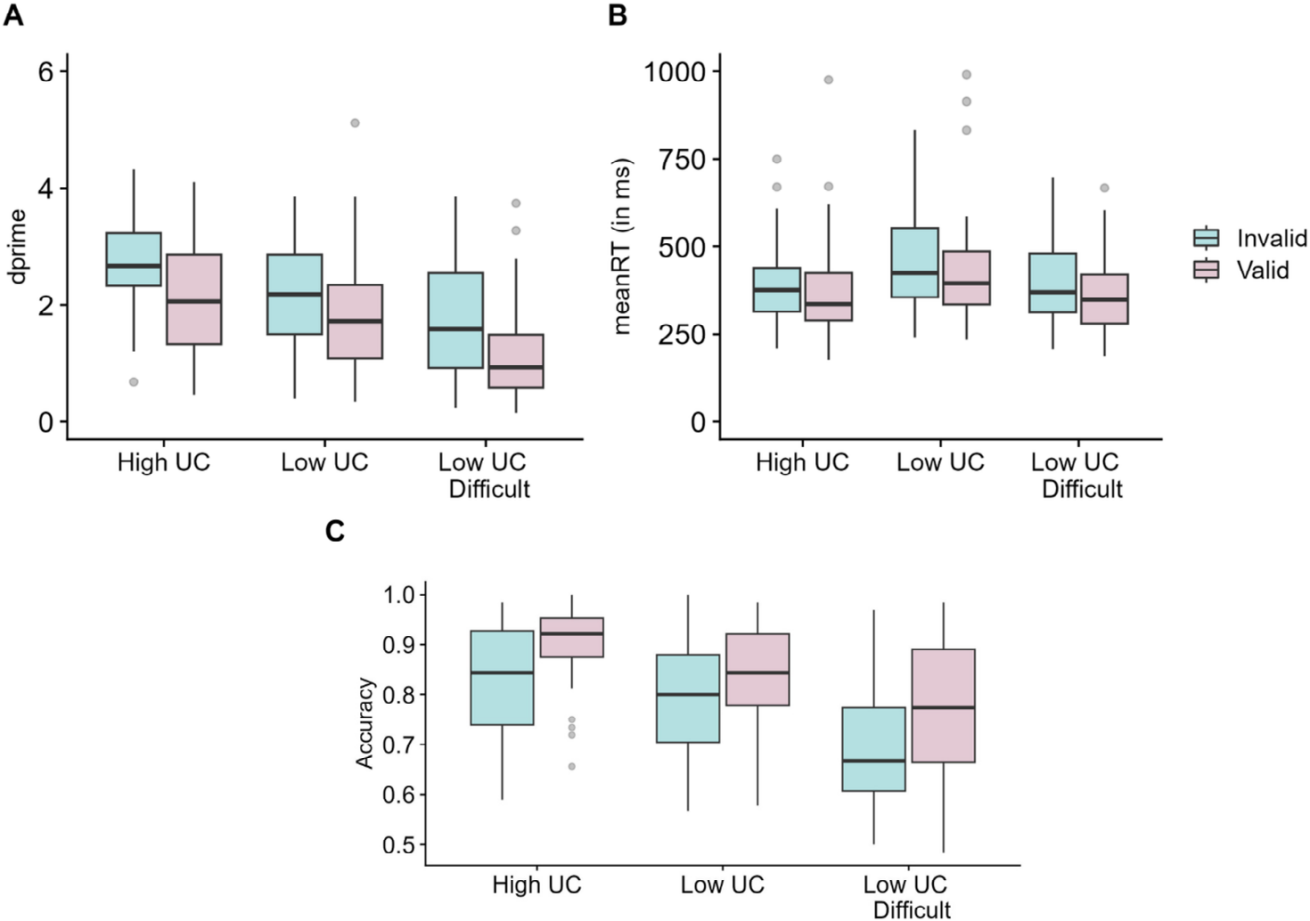
Box plots showing behavioural results of analyses on (A) *d*′, (B) mean RT and (C) Accuracy. The *d*′ on valid trials was higher in the high UC condition compared to the low UC condition. Grey dots represent data points below *Q*1 – 1.5 × IQR and above *Q*3 + 1.5 × IQR, where *Q*1 and *Q*3 represent the first and the third quartile, respectively. IQR, inter‐quartile range.

The behavioural data are shown in Figure 2 and reported in Table 1. Statistical analyses and plots are presented for *d*′', accuracy and mean RT to provide a comprehensive overview of the pattern of results. However, we base our conclusions mainly on *d*′, as *d*′ takes into account response bias and is a more balanced measure compared to accuracy (Stanislaw and Todorov 1999).

**TABLE 1:** *d*′, mean RT and accuracy values for all conditions.

	High uncertainty		Low uncertainty		Low uncertainty difficult	
	Valid	Invalid	Valid	Invalid	Valid	Invalid
*d*′	2.7 (0.8)	2.1 (0.9)	2.2 (0.9)	1.8 (1)	1.7 (1.1)	1.1 (0.9)
Mean RT	376 (155)	414 (167)	393 (184)	434 (201)	429 (139)	489 (174)
Accuracy	0.90 (0.08)	0.83 (0.11)	0.84 (0.1)	0.79 (0.12)	0.77 (0.14)	0.69 (0.12)

There was a significant main effect of UC condition on *d*′ (*F*(2,78) = 38.17, *p* < 0.001, *pes* = 0.49). *d*′ was highest in the high UC condition compared to the low UC (*p* = 0.003) and low UC difficult (*p* < 0.001) conditions. *d*′ was also greater on valid trials compared to invalid trials (*F*(1,39) = 48.19, *p* < 0.001, *pes* = 0.55). There was an almost significant interaction between UC condition and validity (*F*(2,78) = 2.69, *p* = 0.074, *pes* = 0.06). Pairwise comparisons showed higher *d*′ on valid trials on high UC compared to low UC condition (*p* < 0.001) and no difference on invalid trials (*p* = 0.11) indicating greater validity effects in the high UC condition compared to the low UC condition. In contrast, both valid (*p* < 0.001) and invalid trials (*p* < 0.001) differed significantly between low UC difficult and low UC conditions.

The analyses on accuracy also revealed a significant main effect of UC condition, *F*(2,78) = 42.4, *p* < 0.001, *pes* = 0.52. Accuracy was higher in the high UC condition compared to the low UC (*p* = 0.001) and low UC difficult (*p* < 0.001) conditions. Participants were more accurate on valid trials than invalid trials, as indicated by a significant effect of validity, *F*(2,78) = 71.5, *p* < 0.001, *pes* = 0.65. There was no interaction between UC condition and validity, *F*(2,78) = 1.86, *p* = 0.16, *pes* = 0.05.

RTs on valid trials were faster compared to invalid trials as indicated by a main effect of validity (*F*(1,39) = 40.05, *p* < 0.001, *pes* = 0.51). There was also an effect of UC condition (*F*(2,78) = 5.27, *p* = 0.007, *pes* = 0.12) with responses being faster in the high UC condition compared to the low UC difficult condition (*p* = 0.001). There was no difference between high and low UC conditions (*p* = 0.19). Importantly, there was a significant interaction between UC condition and validity (*F*(2,78) = 4.29, *p* = 0.017, *pes* = 0.1). Pairwise comparisons showed greater validity effects in the low UC difficult condition compared to the high UC condition (*p* < 0.05), reflecting the influence of task difficulty on cueing.

### 3.2 Neurophysiological Data

**FIGURE 3:**
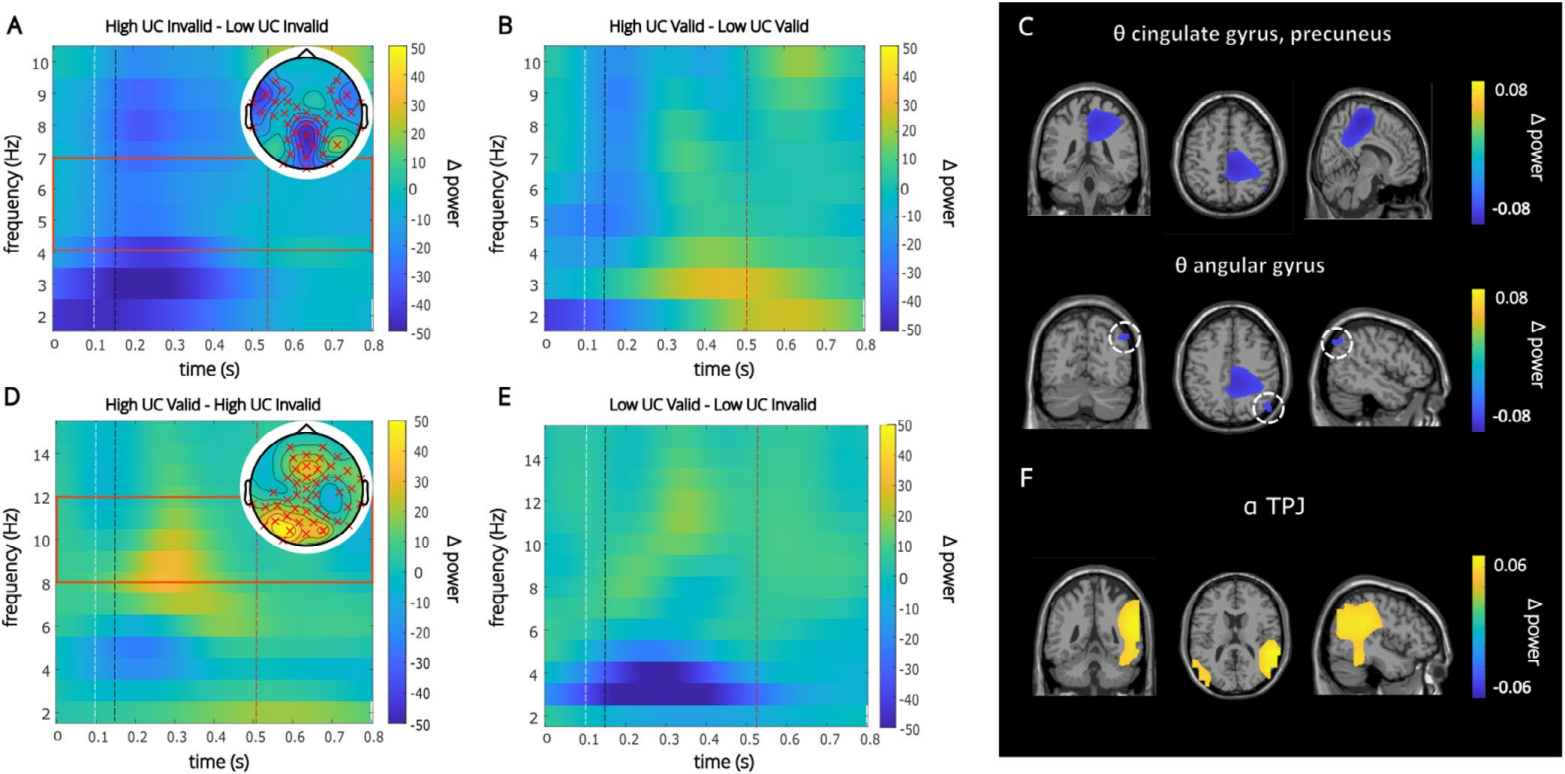
(A) Time–frequency representation showing significant theta band activity. Values represent power differences between high UC and low UC invalid trials. The concentration of negative values indicates greater activity in the low UC invalid condition compared to the high UC invalid condition. (B) Time–frequency representation comparing theta band activity on high UC and low UC valid trials. Topographic plot is not shown since there were no significant differences. (C) Anatomical regions underlying theta activity identified by DICS beamforming and cluster‐based permutation tests. The colour represents power differences between high and low UC invalid conditions. (D) Time–frequency representation showing significant alpha band activity. Power differences between valid and invalid trials of the high UC condition are shown. Positive values indicate greater power on valid trials compared to invalid trials. (E) Time–frequency representation showing no significant differences between low UC valid and invalid trials in the alpha band. (F) Anatomical regions corresponding to alpha band activity under high uncertainty identified by DICS beamforming and cluster‐based permutation tests The time–frequency plots were aligned to the onset of the cue. The white, black and red annotated lines denote the target onset, mask onset and average RT for that condition, respectively. The red rectangles superimposed on (A) and (D) indicate the cluster frequency limits. The red crosses in the topography plots refer to electrodes included in the cluster.

The results of the CBPT and the DICS‐beamforming results are shown in Figure 3. Figures depicting the average alpha and theta power values corresponding to the conditions shown in Figure 3 are given in Figure S1.

In the first level of analyses, we compared valid and invalid trials for each UC condition. The CBPT on the theta‐frequency band (4–7 Hz) at the sensor level in the time window 0–1000 ms after cue onset revealed a significant difference (lower theta power on valid trials compared to invalid trials) for the low UC condition (*p* = 0.03) and the low UC difficult condition (*p* = 0.024, see Supporting Information File Section S3 for visualisation in Data S1). The difference was most pronounced in the parietal and centro‐parietal electrode positions. No significant theta activity differences were observed for the high UC condition. Since there was significant activity in the low UC condition but not in the high UC condition (while comparing valid and invalid trials), we next examined whether the differences between the high and low UC conditions were driven by valid or invalid trials. The difference in the results between high and low UC conditions was driven by differences on invalid trials, as seen by reduced TBA in the high UC invalid condition compared to the low UC invalid condition (*p* = 0.008; Figure 3A). This difference was evident in almost all the channels. There was no difference between the high and low UC valid conditions (*p* = 0.1; Figure 3B). Note that the *p* values reported for the CBPTs were corrected using the Bonferroni method. The uncorrected *p* values were multiplied by the number of comparisons (3) for the differences in validity for each of the three UC conditions. For the two comparisons indicating differences between high and low UC for valid and invalid trials separately, the uncorrected *p* values were multiplied by two. On the source level, DICS beamforming was not performed for the high UC condition since no significant differences were found at the sensor‐level statistics. To examine the source of the different pattern of results for high and low UC conditions, DICS beamforming was performed comparing invalid trials of high and low UC conditions (Figure 3C). This showed activity modulations in the precuneus (BA 7), the paracentral lobule (BA 6) and the middle and posterior cingulate cortex (PCC) (BA 23 and 31). Significant activity modulations were also seen in the IPL including the angular gyrus (BA 39 and 40). DICS beamforming on the negative cluster for the contrast between valid and invalid trials in the low UC condition revealed activity in posterior parietal areas including the postcentral gyrus (BA 3), the precuneus (BA 7) and the paracentral lobule (BA 6). A cluster of voxels was also observed in the middle, inferior and the superior occipital gyrus (BA 19). Significant activity was also seen in the cuneus (BA 17) and mid and posterior cingulum (BA 23 and 31). In the low UC difficult condition, DICS beamforming showed clusters of activity in similar regions.

In the alpha‐frequency band (8–12 Hz), CBPT on the same time window (0–1000 ms after cue onset) with Bonferroni corrections revealed a significant positive difference (valid > invalid, *p* = 0.006; Figure 3D) in the high UC condition. A cluster was observed extending across almost all channel positions. A significant positive difference was also seen in the low UC difficult condition (*p* = 0.006) with the cluster extending across all electrodes (see Supporting Information File S3 in Data S1). In the low UC condition, however, the difference was not significant (*p* = 0.24; Figure 3E) as seen through CBPTs. DICS beamforming comparing valid and invalid trials in the high UC condition (Figure 3F) revealed a cluster of activity in the parietal regions including the precuneus (BA 7), the supramarginal (BA 40) and the angular gyrus (BA 39). Significant activity was also observed in a cluster of voxels in the middle, inferior and superior temporal gyri (BA 21 and BA 22) and the middle and superior occipital gyrus (BA 18 and 19) in this condition. In the low UC difficult condition, a cluster of activity was seen in the same regions, but an additional cluster was observed in the cuneus (BA 17). DICS beamforming was not performed for the low UC condition since only marginally significant differences were found at the sensor‐level statistics.

### 3.3 Neurophysiological Data (nCREANN)

**FIGURE 4:**
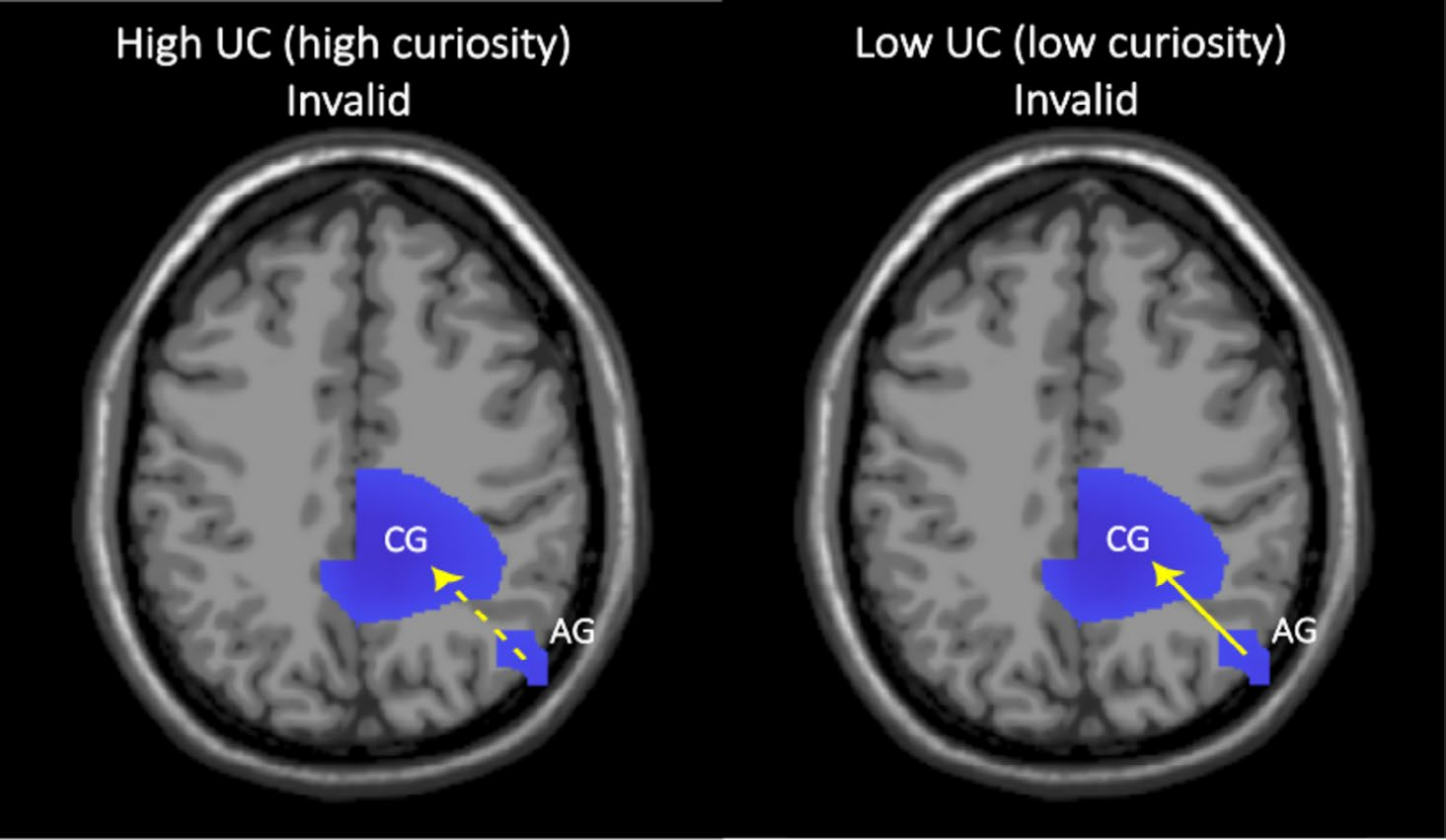
The linear connectivity patterns on the invalid trials of high and low UC conditions. The arrows show the directionality of the connectivity pattern between the two main clusters. There was information flow from AG to CG in both conditions. The dashed arrow indicates a weaker connectivity pattern seen in the high UC condition compared to the low UC condition.

We first report the model validation parameters in Table 2. The results of the nCREANN analysis are shown in Figure 4.

**TABLE 2:** Coefficient of determination (*R* ^2^) and mean square error (MSE) across participants for training and testing data for each condition in the connectivity analyses.

	Training		Testing	
	*R* ^2^	MSE	*R* ^2^	MSE
High UC invalid	0.991 (0.002)	0.03 (0.006)	0.998 (0.002)	0.03 (0.006)
Low UC invalid	0.991 (0.001)	0.01 (0.005)	0.998 (0.002)	0.01 (0.003)

*Note:* The values in brackets denote 1 standard deviation.

The linear connectivity value from AG to CG was higher for the low UC invalid condition (mean = 0.036, SD = 0.018) compared to the high UC invalid condition (mean = 0.03, SD = 0.013), *t* (34) = 2.98, *p* = 0.005 (difference in means = 0.006, SD = 0.012). The reciprocal linear connectivity values from CG to AG did not differ between the two conditions (high UC invalid, mean = 0.029, SD = 0.013; low UC invalid mean = 0.031, SD = 0.018), *t* (34) = 0.35, *p* = 0.727 (difference in means = 0.001, SD = 0.02). A Wilcoxon signed‐rank test showed that the non‐linear connectivity values from AG to CG did not differ between the two conditions either (high UC invalid, median = 0.034; low UC invalid, median = 0.024), *Z* = 0.64, *p* = 0.512. Similarly, the reciprocal non‐linear connectivity values from CG to AG also did not differ between the two UC conditions (high UC invalid, median = 0.024; low UC invalid, median = 0.019), *Z* = 0.28, *p* = 0.777.

## 4 Discussion

We investigated the control processes triggered by uncertainty linked to curiosity. We induced uncertainty in a classic spatial cueing paradigm with peripheral cues (Posner 1980; Prasad et al. 2021) to trigger curiosity. Behavioural results showed that the key interaction between validity and UC condition was only marginally significant. However, planned comparisons based on our earlier study, Prasad and Hommel (2024), showed that *d*′ in the high UC valid condition was higher compared to the low UC valid condition, suggesting greater sensitivity to valid cues in the high UC condition. As expected, the low UC difficult condition led to more errors than the low UC condition, due to increased task difficulty. Since the validity effects in the two conditions were the same, it can be excluded that the effects seen in the high UC condition were merely due to increased task difficulty. Curiosity triggered by uncertainty likely makes people susceptible to task‐irrelevant peripheral cues resulting in greater sensitivity to the cues. This compliments findings of curiosity‐modulated width of an attentional focus (Frings et al. 2019; Gottlieb et al. 2020; Gruber and Ranganath 2019). The greater validity effects observed in the high UC condition emerge because of an increased motivation to explore (Berlyne 1960; Van Lieshout et al. 2020). In contrast to curiosity‐related accounts of uncertainty, control‐related accounts predict that high UC leads to increased TBA (Cavanagh and Frank 2014; Ullsperger et al. 2014; Wu et al. 2021, 2020). TBA has been conceptualised to reflect the ‘need for cognitive control,’ which fits with observations of stronger TBA in situations that require higher cognitive control (Cavanagh and Frank 2014). In our study, however there was no TBA in the high UC condition (as seen in the CBPTs comparing valid and invalid trials in the high UC condition), which suggests that high UC is not always associated with TBA. What implications does this have for the control‐related accounts?

On the one hand, it is possible that the causal chain between uncertainty, TBA and control that these accounts propose (uncertainty → TBA → control) does not exist. However, this chain is consistent with numerous findings that have motivated this account, which would be hard to explain in other ways. On the other hand, it is possible that the proposed chain exists only with some kinds of uncertainty and/or some kinds of control, but not with others. Indeed, the kind of control that Cavanagh and Frank (2014) or Ullsperger et al. (2014) had in mind may represent only one of two types of control that have been discussed recently. Various authors have claimed that individuals can adopt different styles of processing (Beste et al. 2018; Cools 2008; Durstewitz and Seamans 2008; Goschke and Bolte 2014), which vary between persistence (or stability), a style in which information processing is strongly focused on stimuli aligning with current task‐goals and flexibility, a style in which the individual is open to a wide range of information (Hommel 2015; Hommel et al. 2024; Hommel and Colzato 2017). It is interesting to note that persistence has the exact characteristics that the control concept of control‐related approaches to uncertainty have in mind. These approaches are, particularly, interested in uncertainty that is associated with response selection and that can be reduced by focusing on relevant information and the task rules coded in working memory. This is different for curiosity approaches, which are particularly interested in uncertainty regarding the stimulus situation and regarding novel, unexpected information. Dealing with this kind of uncertainty would not benefit from persistence, but from flexibility. If so, control approaches and their proposed link between uncertainty, TBA and control may very well be on track, except that the kind of uncertainty and the kind of control are underspecified. If it would be specified to refer to the chain ‘uncertainty how to respond → TBA → persistence‐type control,’ the account would still be valid. To account for our present findings, however, the chain ‘uncertainty about what is going on → flexibility‐type control’ would be sufficient, without any intervening TBA. In other words, TBA may well be tightly associated with control, but this would only apply to persistence‐type control (or control under a metacontrol bias towards persistence), but not to flexibility‐type control (or control under a metacontrol bias towards flexibility), which we believe underlies the present findings.

Our neurophysiological findings provide a rather systematic picture regarding how curiosity‐inducing uncertainty, flexibility‐based control and integrative information processing go hand‐in‐hand. The modulations in TBA were localised in two distinct clusters: one involving the angular gyrus and the IPL (BA 39 and 40) and the other involving the precuneus (BA 7), para‐central regions (BA 6) and the PCC (BA 23 and 31). The IPL is actively involved in maintaining attention on current goals and detection of salient events so that task goals can be updated accordingly (Husain and Nachev 2007; Malhotra et al. 2009; Singh‐Curry and Husain 2009). Specifically, IPL is involved in directing top‐down goal‐oriented attention to locations in space (Hopfinger et al. 2000). Given that our task involved spatial attention using the cueing task, it is likely that IPL was responsible for deploying goal‐directed attention to target locations. The directed connectivity analyses revealed a pattern of directed communication between the inferior parietal regions and the other large cluster involving the posterior cingulate regions, precuneus and the paracentral regions. This implies that information, likely reflecting top‐down task goals, is transferred from parietal regions to the PCC, precuneus and paracentral regions. This information transfer was weaker for the high UC condition than for the low UC condition. Thus, high curiosity reduces the information transfer of task goals from parietal regions to the PCC, precuneus and the paracentral regions. These latter areas have frequently been associated with TBA‐associated perception‐action integration and action planning (Beste et al. 2023; Domic‐Siede et al. 2021; Nguyen et al. 2021). Especially, the precuneus (BA7) is well‐known to mediate processes of perception‐action integration (Gottlieb 2007; Gottlieb and Oudeyer 2018) and curiosity (Gottlieb and Oudeyer 2018; Van Lieshout et al. 2018). Furthermore, the PCC has been shown to play a key role in the ‘tuning’ of attention (Leech et al. 2011; Leech and Sharp 2014; Pearson et al. 2011; Wilken et al. 2024). The activity in the PCC is increased or decreased depending on the breadth of the attentional focus (narrow vs. broad). The pattern of findings suggests that a reduced impact of task goals (under high curiosity in our study), as communicated from the IPL, leads to the broadening of attentional focus. This makes people susceptible to a wide range of information independent from the current goal. Thus, our findings suggest that the IPL provides weak input regarding the currently active task goals to the PCC and associated areas under high UC, thereby increasing the breadth of the attentional focus. This led to greater processing of task‐irrelevant information. It is worth mentioning that TBA is typically observed in the frontal regions (Cavanagh and Frank 2014) and it can seem surprising that no such activity was seen in our study, especially since the pre‐frontal cortex has been shown to facilitate exploration in the face of uncertainty (Goel 2015; Marinsek et al. 2014). No such effects were found here. The localisation of TBA depends on the precise nature of the task and the type of cognitive processing involved. Since we used a task that manipulates spatial attention and uncertainty, TBA was found to be localised in areas responsible for these mechanisms as mentioned above. Importantly, as mentioned earlier, parietal theta activity is commonly found in studies that involve manipulation of the frequency of stimulus information (Dippel et al. 2016). Thus, these findings are in line with existing theoretical constructs related to attention and uncertainty. The absence of frontal effects along with the absence of theta band activation for the high UC condition can thus be used to fine‐tune our understanding of how different types of uncertainty influence cognitive control. For instance, it is possible that stimulus uncertainty of the type manipulated in our study—not directly associated with response selection—gives rise to a different pattern of activation than response uncertainty which is commonly manipulated in most studies. Further studies are required to tease apart these differences both at the behavioural and the neurophysiological level.

Modulations of TBA aside, we also expected curiosity to modulate earlier stages of processing like attentional filtering, which is reflected by ABA (Herrmann and Knight 2001; Klimesch 2011; Klimesch et al. 2011). For ABA, we observed positive clusters indicating stronger activity on valid trials compared to invalid trials. It is possible to question if this pattern of activation reflects low‐level sensory processing. If that were the case, we should have seen similar differences between valid and invalid trials across all uncertainty conditions since low‐level sensory effects should presumably be unaffected by higher‐order mechanisms such as the uncertainty manipulation. Interestingly, ABA differences between valid and invalid trials were specific for the high UC condition, but not for the low UC condition. ABA is said to reflect a general inhibitory gating mechanism by which task‐irrelevant/distracting information is suppressed through which the access to information for task‐relevant behaviour is controlled (Jensen and Mazaheri 2010; Klimesch 2012; Konjusha et al. 2023; Rihs et al. 2007; Yu et al. 2024). In line with this, higher ABA should have been seen on invalid trials since the peripheral cues are task‐irrelevant on these trials. But we see the opposite result suggesting that suppression of task‐irrelevant information on invalid trials was weaker under high UC. Thus, high UC makes the inhibitory gating mechanism less effective reflecting reduced ABA on invalid trials (see Prochnow et al. 2022; Pscherer et al. 2023 for similar findings in ABA). The ABA under high UC was localised in the temporo‐parietal junction (TPJ) including the supramarginal (BA 40) and angular gyrus (BA 39) which is in line with TPJ's role in reorienting attention to salient stimuli in a contextually relevant manner (Behrmann et al. 2004; Geng and Vossel 2013). Thus, it seems likely that, under high UC, TPJ was responsible for making contextual adjustments to the attentional system and reoriented participants' attention to the peripheral cues more strongly on invalid trials.

It is possible to question whether the task used in our study, which is typically used to measure attention control, truly engages cognitive control in the way traditional control tasks (e.g., Go No‐go) do. However, on the invalid trials of the Posner cueing task, participants are required to suppress the prepotent response of orienting to the cue and instead attend to the target location. It is reasonable to assume that this mechanism involves cognitive control. Further, whether there are clear differences between attention and cognitive control depends on whom we ask. Several models consider attention as part of cognitive control or even use them interchangeably (Braver 2012; Engle and Kane 2004). The authors of this paper also strongly believe that it is important to move beyond paradigmatic research, where certain mechanisms are explicitly tied only to specific tasks (Frings et al. 2024; Hommel 2020). If cognitive control is understood as a mechanism that regulates goal‐directed behaviour, then it must be possible to engage cognitive control in any task that involves ignoring irrelevant information and selecting task‐relevant responses, such as the Posner cueing task.

One unexpected finding from this study was that there were more accurate responses in the high UC condition compared to the low UC condition. This contradicts earlier findings regarding the main effect of uncertainty on accuracy in studies with a similar task design (Prasad and Hommel 2024; Shiu and Pashler 1994). It is worth noting that these differences in accuracy were driven by differences in valid trials. Specifically, accuracy in the high UC valid condition was higher compared to the low UC valid condition, resulting in overall higher accuracy in the high UC condition. Since valid cues reliably predict the target location and therefore help to respond to the target, this suggests that the cues were processed more when the uncertainty was high. The neurophysiological findings were also in line with this argument that high UC is associated with a flexible‐type control, as indicated by the lack of significant TBA under high UC.

We also did not observe faster responses in the low UC condition compared to the high UC condition as seen in Prasad and Hommel (2024) which is a puzzling anomaly. However, in all these studies, there was an interaction between uncertainty and validity either on *d*′ or mean RTs. The key difference is that in our study, there were differences between high and low UC in valid trials, whereas in the previous two studies, the effect was driven by differences in invalid trials. Thus, it is not clear if uncertainty primarily influences valid trials invalid trials or both. It is important to note that the interaction between uncertainty and validity was only marginally significant for *d*′ in the current study, which is in contrast to our earlier study. This is unlikely due to an inadequate sample size, as we had the same number of participants in both studies. All aspects of the design were also the same. We have already observed this effect in three separate experiments with 40 participants each in our earlier study. In spite of the weak interaction, planned comparisons revealed highly significant differences between high and low UC conditions on valid trials in the current study. Finally, there were clear differences at the neurophysiological level. It is known that even with true reliable effects, depending on the power, the *p* values can vary over several studies such that they are not always below the threshold of significance (Greenland et al. 2016; Hung et al. 1997). This could be one of the reasons for the weak interaction effects observed in our study, which can only be confirmed through more research using this paradigm.

Another point worth mentioning is that curiosity was only indirectly implicated in our study through its theoretical links with uncertainty, which could be considered a limitation of the current study. Curiosity is typically measured through self‐report scales (e.g., Collins et al. 2004; Kashdan et al. 2018), but objectively validating the induction of a curious state is not straightforward. Some studies have inferred curiosity through decisions to sample more information (Gottlieb et al. 2020; Van Lieshout et al. 2020). Participants are more likely to seek additional information before responding when their subjective confidence is not high (Desender et al. 2018; Nicki 1970). Even though we did not evaluate subjective confidence in our study, high UC is often associated with lower confidence (Cohanpour et al. 2024; Metcalfe et al. 2023). Thus, we have interpreted greater cueing effects under high UC (and low confidence) to reflect the decision to seek more information from the cues (Beesley et al. 2015). But we do acknowledge that it is important for future research to verify the conclusions of this study using more direct tests of confidence and curiosity. It is also important to replicate the study's key neurophysiological finding (absence of TBA in the high UC condition) using larger sample sizes while including more detailed demographic measures such as IQ.

In conclusion, we show that some kinds of uncertainty make people attend to seemingly irrelevant cues, which happened in the absence of TBA. Effective connectivity analyses also showed weaker connections between inferior parietal regions and the PCC under high UC. These (from a classical control view) counter‐intuitive results indicate that uncertainty does not always trigger TBA. Activity in the alpha band and the corresponding neuroanatomical regions provide further evidence for the role of uncertainty in attention control. Taken altogether, we conclude that the widely assumed causal chain from uncertainty to TBA to control is not as general as has been claimed. Our findings suggest that the actual chain depends on the kind of uncertainty and the kind of control that is involved. Whereas response‐related uncertainty may indeed trigger TBA, resulting in higher persistence‐type control, stimulus‐related uncertainty may not trigger TBA, resulting in higher flexibility‐type control. Thus, the connection between uncertainty and cognitive control depends on the metacontrol implications of the particular kind of uncertainty being involved.

## Author Contributions

**Seema Prasad:** conceptualization, methodology, software, formal analysis, visualisation, funding acquisition, investigation, writing – original draft, writing – review and editing. **Nasibeh Talebi:** software, formal analysis, visualisation. **Paul Wendiggensen:** software, formal analysis, validation. **Moritz Mückschel:** software, formal analysis, validation. **Bernhard Hommel:** conceptualization, methodology, supervision, writing – original draft, writing – review and editing. **Christian Beste:** conceptualization, methodology, supervision, resources, project administration, funding acquisition, writing – original draft, writing – review and editing.

## Ethics Statement

This research was approved by the Ethics Committee of TU Dresden.

## Conflicts of Interest

The authors declare no conflicts of interest.

## Supporting information


**Data S1:** Supporting Information.

## Data Availability

The data that support the findings of this study are openly available in OSF at https://osf.io/gn3vf/?view_only=d3fd38707dac45bb8e885a77f30b1230.
