# Supplementary material for: Uncertainty, Cognitive Control and Theta‐Band Activity: A Relationship That Depends on Metacontrol Requirements
Source: Hum Brain Mapp. 2025 Sep 23;46(14):e70333. doi: 10.1002/hbm.70333 (PMC12455151; doi:10.1002/hbm.70333)
Supplement: Supplementary file 1 — Data S1: Supporting Information. [file HBM-46-e70333-s001.docx]

**Supplementary material**

**Uncertainty, cognitive control, and theta-band activity: a relationship that depends on metacontrol requirements**

Seema Prasad, Nasibeh Talebi, Paul Wendiggensen, Moritz Mückschel, Bernhard Hommel, Christian Beste

**
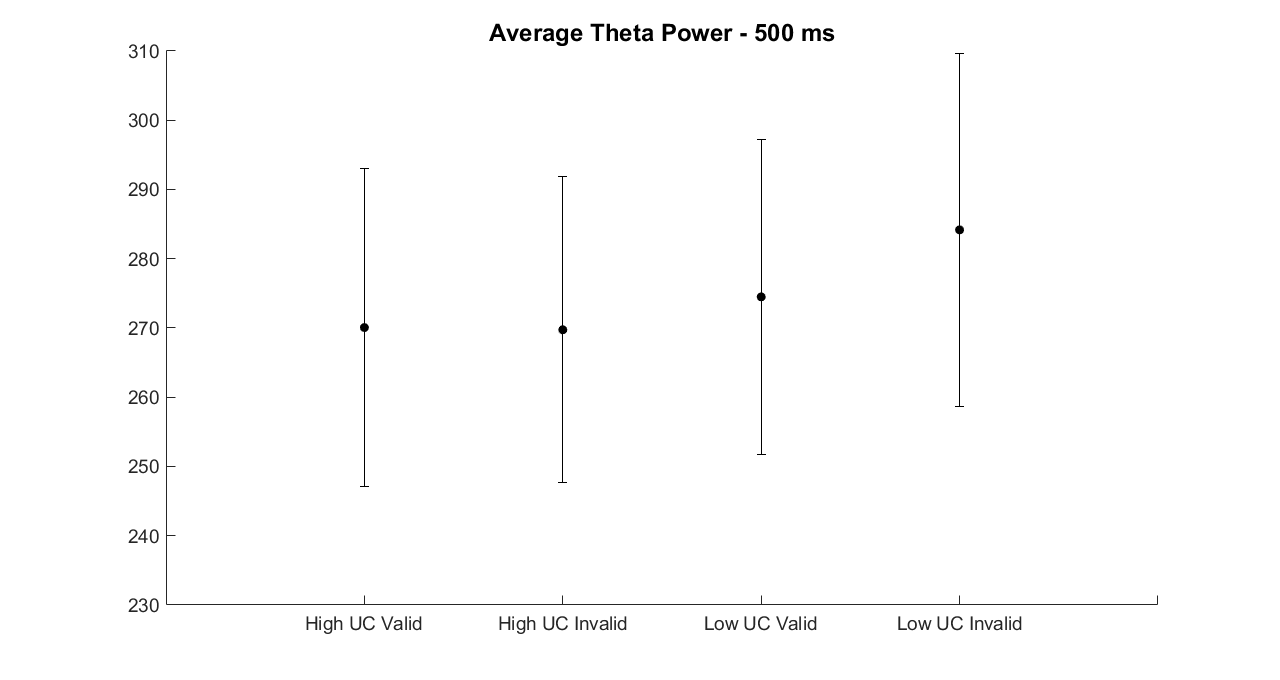

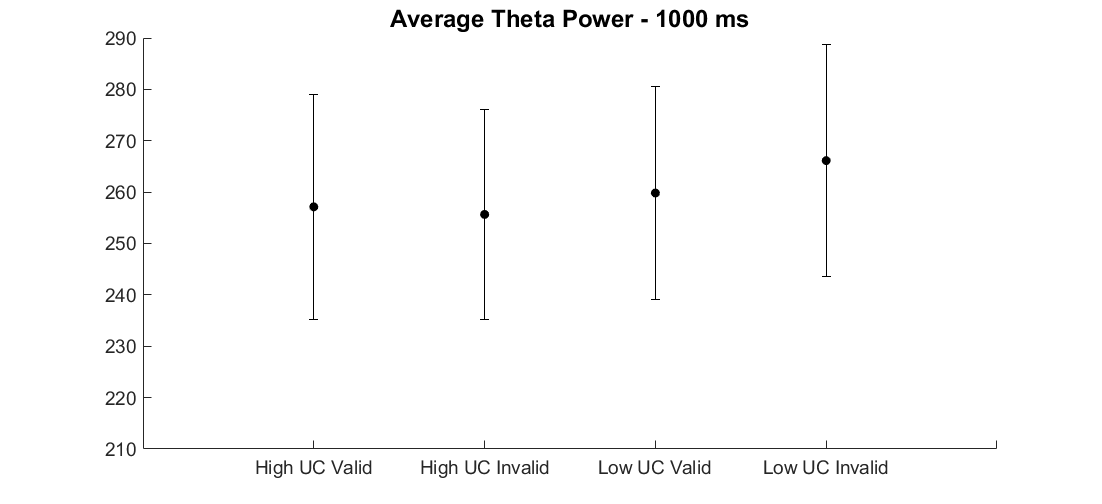
S1. Average Theta and Alpha power for each of the four main conditions**

The average values were calculated by averaging the power over all channels and over time. The average power for the time window 0 – 1000 ms and 0 – 500 ms are given below for theta and alpha band activity. It is important to note that the pattern of results in these graphs may not necessarily match the results from the cluster-based permutation tests (CBPT) performed on time-frequency data reported in the manuscript (and in Figure 3). This is because CBPT conducts multiple tests at each time point and spatial location from which clusters of activity are found at specific channel locations and time periods. In contrast, the power values presented below in the figures are simple grand averages across all electrodes which do not adequately capture the complexity of the data.


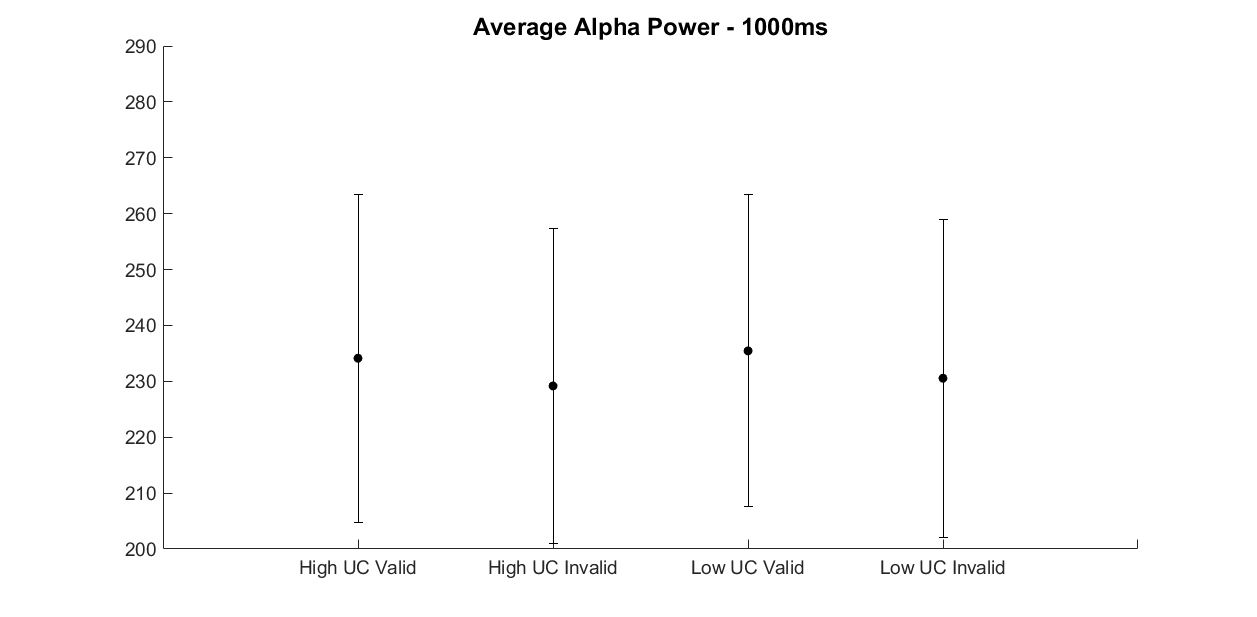

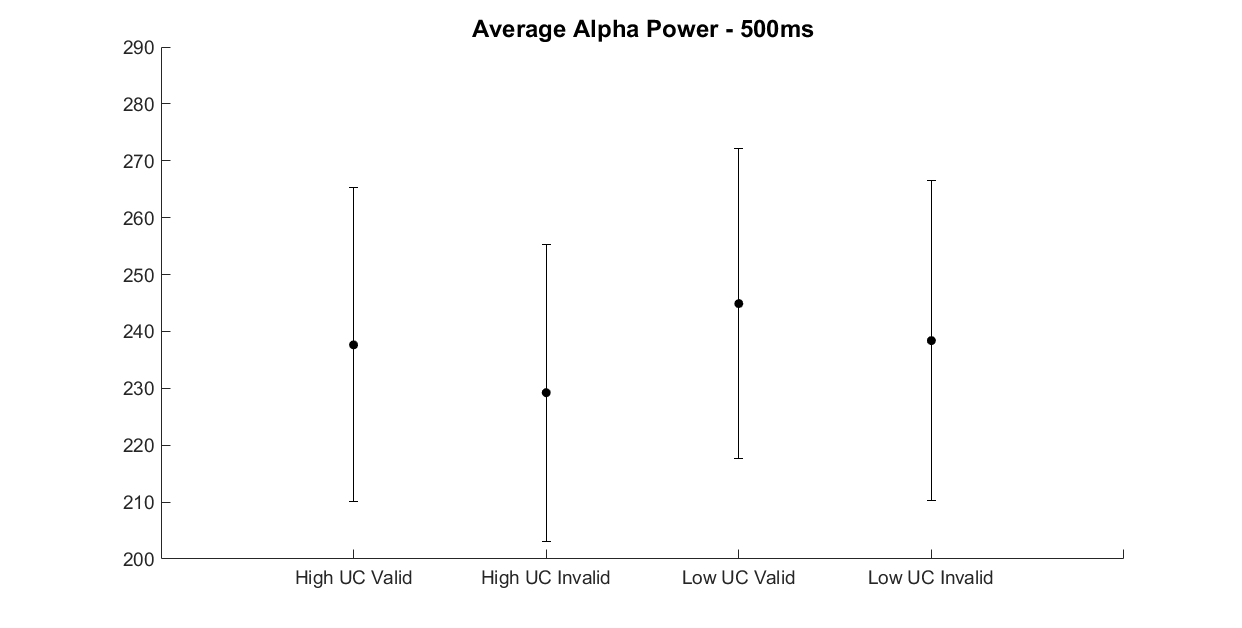


Figure 1. Average theta and alpha power for 0 to 500 ms and 0 to 1000 ms durations

**S2. Statistical analyses on Accuracy data**

Repeated measures ANOVA on Accuracy with Condition (high UC, low UC, low UC difficult) and Validity (Valid, Invalid) revealed a significant main effect of Condition, *F* (2, 78) = 42.4, *p* < 0.001, *pes* = 0.52 and Validity, *F* (1, 39) = 71.5, *p* < 0.001, *pes* = 0.65. The interaction between the two did not reach significance, *F* (2, 78) = 1.86, *p* = 0.16, *pes* = 0.05.

**S3. Time-frequency plots for theta and alpha band activity in the low UC difficult condition**


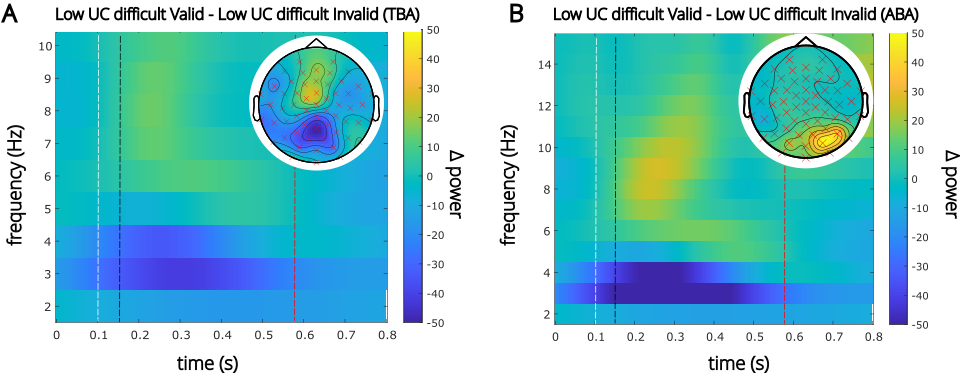


*Figure 2. Time-frequency representation showing significant TBA (A) and ABA (B) for the low uncertainty difficult condition comparing valid and invalid trials. The time-frequency plots were aligned to the onset of the cue. The white, black and red annotated lines denote the target onset, mask onset and average RT for that condition, respectively.*
